# The impact of reconstruction algorithms and time of flight information on PET/CT image quality

**DOI:** 10.1515/raon-2015-0014

**Published:** 2015-08-21

**Authors:** Alen Suljic, Petra Tomse, Luka Jensterle, Damijan Skrk

**Affiliations:** 1 Faculty of Health Sciences, University of Ljubljana, Slovenia; 2 Department for Nuclear Medicine, University Medical Centre Ljubljana, Slovenia; 3 Slovenian Radiation Protection Administration, Ljubljana, Slovenia

**Keywords:** time of flight, PET/CT, point spread function, reconstruction algorithm, image quality

## Abstract

**Background:**

The aim of the study was to explore the influence of various time-of-flight (TOF) and non-TOF reconstruction algorithms on positron emission tomography/computer tomography (PET/CT) image quality.

**Materials and methods.:**

Measurements were performed with a triple line source phantom, consisting of capillaries with internal diameter of ∼ 1 mm and standard Jaszczak phantom. Each of the data sets was reconstructed using analytical filtered back projection (FBP) algorithm, iterative ordered subsets expectation maximization (OSEM) algorithm (4 iterations, 24 subsets) and iterative True-X algorithm incorporating a specific point spread function (PSF) correction (4 iterations, 21 subsets). Baseline OSEM (2 iterations, 8 subsets) was included for comparison. Procedures were undertaken following the National Electrical Manufacturers Association (NEMA) NU-2-2001 protocol.

**Results:**

Measurement of spatial resolution in full width at half maximum (FWHM) was 5.2 mm, 4.5 mm and 2.9 mm for FBP, OSEM and True-X; and 5.1 mm, 4.5 mm and 2.9 mm for FBP+TOF, OSEM+TOF and True-X+TOF respectively. Assessment of reconstructed Jaszczak images at different concentration ratios showed that incorporation of TOF information improves cold contrast, while hot contrast only slightly, however the most prominent improvement could be seen in background variability - noise reduction.

**Conclusions:**

On the basis of the results of investigation we concluded, that incorporation of TOF information in reconstruction algorithm mostly affects reduction of the background variability (levels of noise in the image), while the improvement of spatial resolution due to incorporation of TOF information is negligible. Comparison of traditional and modern reconstruction algorithms showed that analytical FBP yields comparable results in some parameter measurements, such as cold contrast and relative count error. Iterative methods show highest levels of hot contrast, when TOF and PSF corrections were applied simultaneously.

## Introduction

The first advantages of time-of-flight (TOF) technique for positron emission tomography (PET) were presented in the early 1980s. The idea of using the TOF information in PET was implemented in the first generation of the TOF PET scanners using crystal materials with relatively low time resolution.^1^,^2^ TOF PET is characterized by a better trade-off between contrast and noise in the image.^3^–^7^ This property is used in more challenging clinical conditions, allowing shorter examinations at lower count rates, successful scanning of larger patients, clearer characterization of low uptake areas and visualization of smaller lesions.^8^–^12^ Accompanied with the specific point spread function (PSF) correction it produces images with high image quality.^13^,^14^ Current endeavours in research are mainly oriented towards improving the time resolution. Recent study of TOF PET using Cherenkov light reached coincidence resolution of 71 ps full width at half maximum (FWHM).^15^

Karp *et al.* investigated the benefits of TOF correction in experimental phantoms and concluded that TOF correction leads to a better contrast-to-noise trade-off than non-TOF. They pointed out that complete impact of TOF should not be investigated in terms of a simple sensitivity gain improvement.^10^ Akamatsu *et al.* investigated the effect of PSF and TOF corrections on PET/CT image quality with different reconstruction parameters and count rates. They determined that PSF and TOF corrections slightly improve contrast and background variability.^16^

Review of the literature indicates that image quality improvement is expected with incorporating TOF correction in reconstruction algorithm.^16^,^17^ The aim of present research was to evaluate image quality parameters using different reconstruction algorithms, altering phantoms, activity concentration ratios and regions of interest with special focus on TOF information impact.

## Materials and methods

All measurements were performed at the Department of Nuclear Medicine, University Medical Centre Ljubljana on Biograph mCT PET/CT scanner, manufactured by Siemens. Scanner combines a 128-slice CT and patented lutetium oxyorthosilicate (LSO) PET system for whole body imaging with included TOF technique. The gantry aperture is 78 cm wide and the tunnel length is 136 cm. This model of PET/CT scanner has incorporated PET Syngo VG30 software. The study was performed on a triple line source phantom and on the Jaszczak phantom. To insure adequate comparison with presented values in literature, the measurements in both phases were performed according to National Electrical Manufacturers Association (NEMA) NU-2-2001 standard.^18^

### Measurement of spatial resolution

**FIGURE 1.**
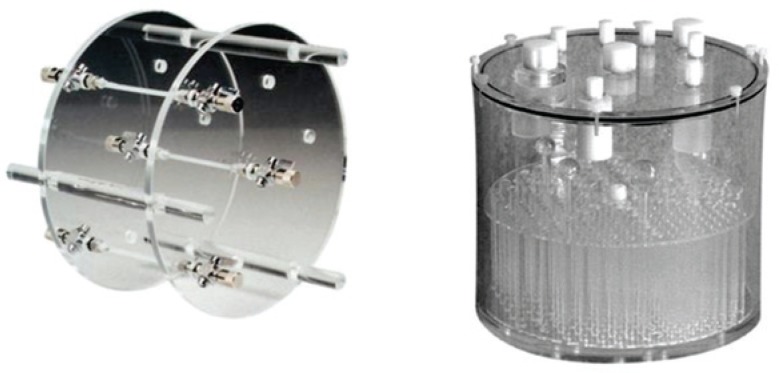
Triple line insert for spatial resolution measurements and Jaszczak phantom for measurements of described image quality parameters.

Spatial resolution was evaluated following the NEMA NU-2-2001 standard using a triple line source of ^18^F (activity concentration 7 MBq/ml). Triple line insert phantom (Triple Line Insert, Data Spectrum Co.) was used to obtain three 1 mm diameter parallel lines of tracer material spaced 7.5 cm apart (Figure 1). The total activity was low enough to keep dead time losses and the ratio of randoms to total events below 5%, as suggested by the protocol.^18^–^20^ The acquisition of data was performed with 4.1 ns coincidence window and 12% energy window. The measurements were performed with phantom centre positioned at three locations within PET ring; (1) *x* = 0 and *y =* 1 cm (to avoid the exact centre of the scanner where the sampling density of lines of response may be very high), (2) x = 0 and y = 10 cm, and (3) x = 10 and y = 0 cm. The acquired data was reconstructed using analytical filtered back projection (FBP), iterative ordered subsets expectation maximization (OSEM) (4 iterations, 24 subsets) and iterative True-X (4 iterations, 21 subsets) which incorporates PSF correction. All images were reconstructed into a matrix of 400×400 with a 1 mm pixel size. All reconstructions included a Gaussian post-filter of 4 mm FWHM. Values of intrinsic spatial resolution – FWHM_int_ were calculated according to equation in Skreting *et al*.^21^, in which FWHM_eff_ is the FWHM of profile measured on the reconstructed image and FWHM_filter_ is the width of the Gaussian reconstruction filter.

$${\mathrm{FWHM}}_{\mathrm{int}}=\sqrt{{\mathrm{FWHM}}_{\mathrm{eff}}^{2}-\mathrm{FWH}M_{\mathrm{filter}}^{2}}$$

### Measurements of image quality parameters

Due to the complex interplay of different aspects of imaging system, it is desirable to be able to compare the image quality of different systems using a standardized imaging situation that simulates a clinical imaging condition. In order to evaluate the quality of the image simulating a clinical whole body acquisition, Jaszczak phantom PET/FL-X2/P (Data Spectrum Co.) was used (Figure 1). The phantom consists of the lid, the body of phantom and the cold spheres insert. Lid has seven little cylinders, six of which are hollow with external diameters of 8 mm, 12 mm, 16 mm and 3 cylinders with diameters of 25 mm. The seventh cylinder is solid and simulates bone on reconstructed image (teflon). The body of the phantom holds the volume of ∼ 6 L. The cold insert holds spheres with diameters of 9.5 mm, 12.7 mm, 15.9 mm, 19.1 mm, 25.4 mm and 31.8 mm.^18^ The four smallest cylinders (8 mm, 12 mm, 16 mm and 25 mm) and the body of the phantom were filled with a radioactive solution with three different cylinder-to-background activity concentrations of 2:1 (48 kBq/ml:24 kBq/ml), 4:1 (88 kBq/ml: 22 kBq/ml) and 8:1 (144 kBq/ml:18 kBq/ml) in 3 sequential acquisitions. The coincidence and energy window settings remained the same as in spatial resolution measurements. The two larger cylinders were filled with water and air, respectively. The phantom was placed so that the spheres were in the same transversal plane, coinciding with the central plane of the scanner. Corrections (intensity normalization, scatter and random events, dead time losses and attenuation with the CT) were applied in the reconstruction into a matrix of 512 × 512 with 1.6 mm pixel size and Gaussian post-filter of 4 mm of FWHM. We used different image reconstruction algorithms - analytical filtered back projection (FBP), iterative OSEM (4.24) and iterative True-X with PSF correction (4.21). TOF information was alternately incorporated in each reconstruction algorithm. Baseline iterative OSEM (2.8) reconstruction method was added for comparison. Evaluation of image quality was performed by calculation and observation of the following image parameters: percentage of contrast of hot cylinders and cold spheres, percentages of background variability (in the vicinity of hot cylinders and cold spheres) and percentage of relative count error.

Percentage of the contrast of the hot cylinders and cold spheres was determined from the average counts in the cylinders and spheres, as well as in the background which were measured in regions of interest (ROI) with the same size as the cylinders or spheres. Contrast Q_H,j_ for cylinder j was calculated by:

$$Q_{H,j}=\frac{\frac{C_{H,j}}{C_{B,j}}-1}{\frac{a_{H}}{a_{B}}-1}\times 100\%$$

where C_H,j_ is the average counts in ROI for the cylinder *j*, C_B,j_ is the average of background ROI, a_H_ is the activity concentration in cylinders and a_B_ is the activity concentration in the background (both a_H_ and a_B_ were measured in dose calibrator before PET acquisition). Contrast of spheres Q_C,j_ for each cold sphere j was calculated by:

$$Q_{C,j}=\left(1-\frac{C_{C,j}}{C_{B,j}}\right)\times 100\%$$

where C_C,j_ is the average counts in the ROI for sphere *j* and C_B,j_ is the average of the background ROI counts for sphere *j*.

Percentage of background variability was calculated as the ratio between the standard deviation and the mean value in 12 randomly placed concentric ROI in the background that were at least 15 mm away from any cylinder, sphere or the edge of the phantom. The sizes of ROI corresponded to the diameters of the spheres. The percent background variability N_j_ for sphere j is calculated as:

$$N_{j}=\frac{SD_{j}}{C_{B,j}}\times 100\%$$

where SD_j_ is the standard deviation of the background ROI counts for sphere *j* and C_B,j_ is the average of the background ROI counts for sphere *j*.

The relative count error that evaluates the accuracy of the scatter and attenuation corrections was determined as the average of the relative count errors in 2 planes. This was obtained as the ratio between the mean value of counts in a circular region (of 22 mm or 25 mm in diameter, positioned in the air filled cylinder) and the mean background value (evaluated in 12 regions of the same size). We expected the contribution of scatter and attenuation error that was evaluated for air to be most prominent in the voxels closest to the background which also includes 1.5 mm plastic cylinder wall. Besides estimating the value for purely air medium, we found that it was important to take into account the cylinder wall for comparison. Therefore 2 diameters of ROI were used, including and excluding the cylinder wall (22 mm and 25 mm). The residual error in scatter and attenuation corrections ΔC_air,i_ for each slice i was calculated as:

$$\Delta C_{\mathrm{air},i}=\frac{C_{\mathrm{air},i}}{C_{B,i}}\times 100\%$$

where C_air,i_ is the average counts in the air filled cylinder ROI and C_B,i_ is the average count of the background ROI for slice i.

## Results

Results are presented in the same order as they were presented theoretically in the previous chapter. Spatial resolution results, measured on triple line insert, are followed by contrast, background variability and relative count error results, measured on Jaszczak phantom.

### Spatial resolution

The spatial resolution of a system represents its ability to distinguish between two points after image reconstruction. The measurement of spatial resolution characterizes the shape of the reconstructed point spread function at the FWHM level. Such measurement allows a reliable evaluation of scanners, taking into account the variation in spatial resolution with radial distance. The data are taken at low counting rates, so that potential event pileup is not encountered. Table 1 summarizes the results of the spatial resolution measured in air on PET/CT scanner.

### Hot and cold contrast

**FIGURE 2.**
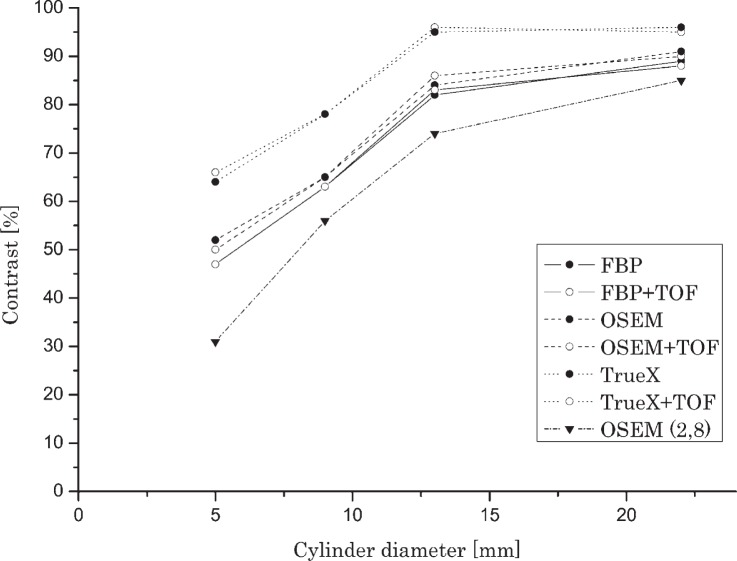
Hot contrast in relation with cylinder diameter and reconstruction method (activity concentration ratio 8:1).

**FIGURE 3.**
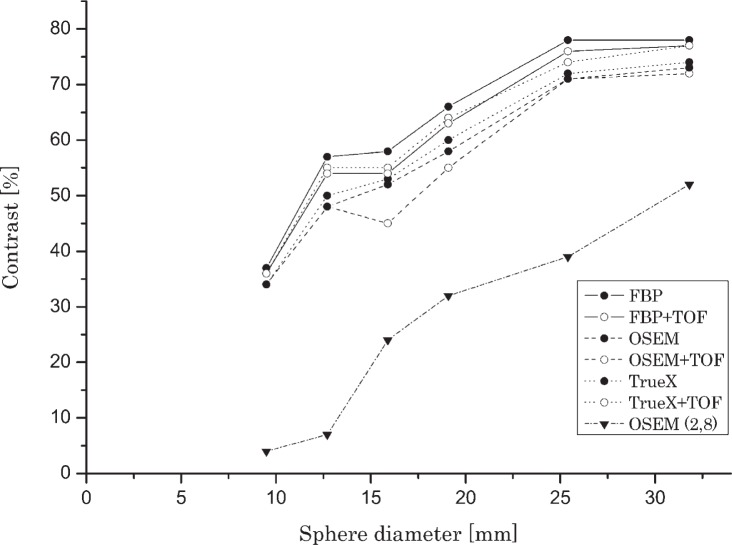
Cold contrast in relation with sphere diameter and reconstruction method (activity concentration ratio 1:8).

The measured parameters of image quality depend on reconstruction algorithm used. Figures 2 and 3 show the response of observed reconstruction methods in relation to cylinder or sphere diameter and activity concentration ratio between cylinders or spheres and background. Presented activity concentration ratios were chosen for best representation of the results. The iterative algorithm True-X with TOF correction displayed the best results of hot contrast. Slightly lower levels of contrast were shown (with smallest spheres) with iterative OSEM (4.24), followed closely by analytical FBP. Algorithms with incorporated TOF correction displayed slightly better results as their non TOF counterparts. Iterative algorithm True-X with TOF correction displayed the best result of cold contrast, followed closely by analytical FBP. Baseline iterative OSEM (2.8) showed the lowest hot and cold contrast. TOF information had higher impact with cold contrast performance in comparison with hot contrast performance. For all sizes of cylinders and spheres, the hot contrast increased with iterative reconstruction methods, however in cold contrast traditional FBP showed slightly better results, especially for larger spheres.

### Background variability

**FIGURE 4.**
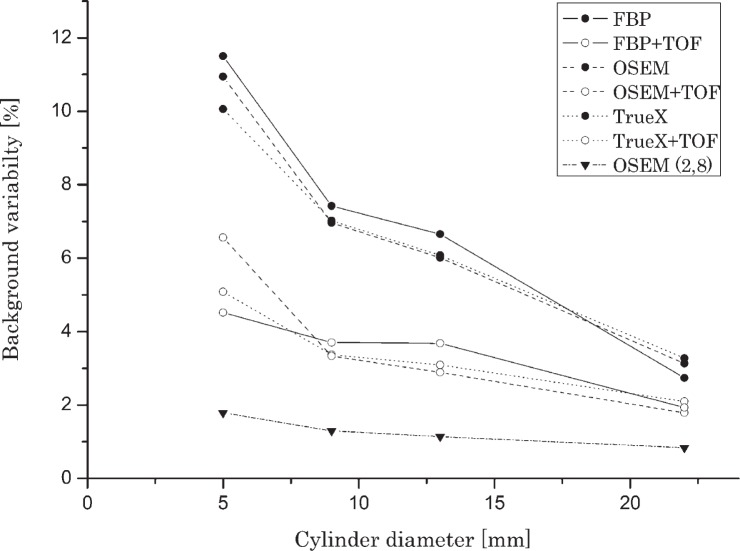
Background variability in relation with cylinder diameter and reconstruction method (activity concentration ratio 2:1).

**FIGURE 5.**
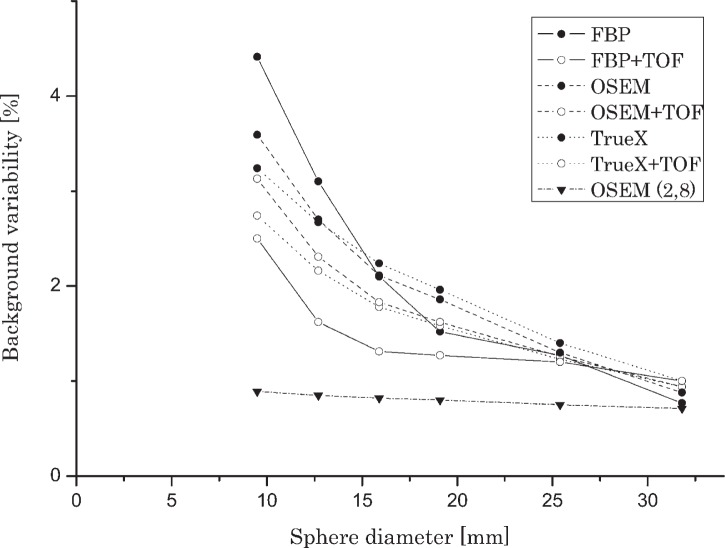
Background variability in relation with sphere diameter and reconstruction method (activity concentration ratio 1:2).

Figures 4 and 5 show variability of background for all reconstruction methods and all (cylinders or spheres) diameters. The TOF correction significantly reduced background variability – up to 50% for all reconstruction algorithms especially with the smallest diameter spheres. The measurement of background variability in the vicinity of cold spheres is not foreseen in the NEMA protocol; however our research shows that the values of background variability in the vicinity of hot cylinders and cold spheres differ by a factor of three. The impact was more prominent for cylinders and spheres of smaller diameters. Baseline OSEM (2.8) produced images with lowest values of background variability, or in other words, highest uniformity and lowest noise levels.

### Relative count error

Relative count errors for various reconstruction methods and activity concentration ratios are presented in Table 2. We found some difficulties with positioning ROI in areas with low concentration ratio, since there is a cylinder wall around observed air medium in the background with higher specific activity, which has to be taken into account. This issue was not addressed in standard protocol but as we found it important, we chose to compare measurements with and without 1.5 mm thick cylinder wall accounted in ROI measurements (22 mm and 25 mm). The measurements were made in cylinder filled with air as opposed to the measurements made in lung insert with fixed density 0.3 g/cm^3^, cited by NEMA protocol and other authors.^5^,^17^,^18^,^22^

## Discussion

The spatial resolution measurements show that PSF modelling successfully counteracts the parallax error and is responsible for spatial resolution improvement throughout field of view. The results are in line with results of other authors and confirmed the accuracy of used methods. Slight misalignments of a line source with the scanner axis leads to degraded resolution compared with that measured with a point source. The spatial resolution measured with a point source, therefore, can be expected to be slightly better than that determined with a line source (approximately few tenths of a millimetre).^23^ The objective of the image quality test was to produce images simulating whole body scans with hot and cold lesions. The measurements were extended to include the contrast ratios 2:1, 4:1 and 8:1 between the hot cylinders and background, in addition to evaluation of different modern and especially traditional reconstruction algorithms, not contemplated by NEMA protocol. Results of hot and cold contrast show that incorporation of TOF information only marginally improves contrast recovery. Best results were achieved with iterative reconstruction algorithm incorporating PSF modelling-True-X with TOF information. Baseline OSEM (2.8) produced images with lowest contrast ratio in comparison with other reconstruction methods. The most important improvement of contrast was obtained with the incorporation of PSF in the reconstruction, while TOF having lower impact.

Results of background variability showed that TOF information has the most profound impact. Incorporation of TOF information resulted in up to 50% reduction of background variability with all observed reconstruction algorithms. In clinical application the improvement of background variability means lower patient dose or reduction of the imaging time at the same level of image noise. The background variability in the vicinity of hot inserts was higher up to three times compared to background variability in the vicinity of cold inserts. Best results were achieved with baseline reconstruction algorithm OSEM (2.8) where we reconstructed images with the lowest levels of noise. This algorithm was included in this research because it was the usual method of reconstruction in the previous generation of PET tomographs.^16^ It is important to understand that the background variability parameter presents not only statistical noise but also non uniformities in the image which arise from inaccurate attenuation correction or poor convergence during iterative reconstruction. The background variability does not reflect noise correlations or streak artefacts in the image.^23^

The results of relative count error which provides information of accuracy of attenuation and scatter corrections show, that incorporation of TOF reconstruction in most cases improved (decreased) relative count error, especially at higher activity concentration ratios. Best results were surprisingly obtained with FBP with incorporated TOF correction. The use of PSF correction does not show the improvement of the results, already obtained with TOF correction. The results were similar in evaluation of the cold contrast and the error in the air, since the radioactivity is measured in an image segment in which there is no activity and only the medium varies. The different measurements of relative count error show that the differences between measurements with internal diameter sized ROI and external diameter sized ROI can be as high as 10%.

**FIGURE 6.**
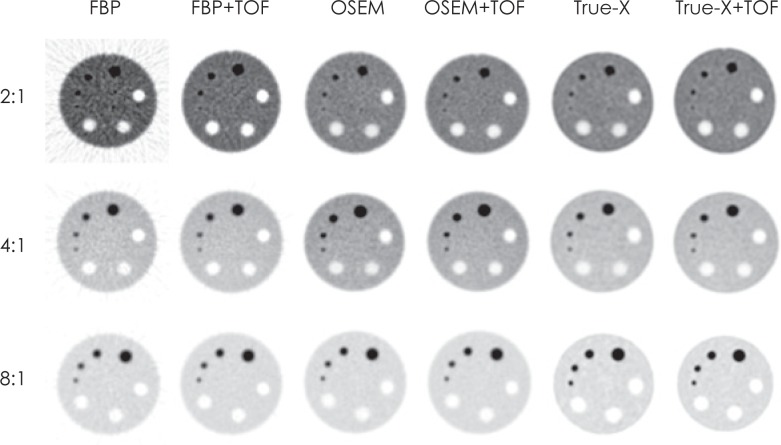
Visual assessment of image quality according to the reconstruction method and activity concentration ratio. FBP = filtered back projection; FBP+TOF = filtered back projection with incorporated time of flight information; OSEM = ordered subsets expectation maximization; OSEM+TOF = ordered subsets expectation maximization with incorporated time of flight information; True-X = iterative reconstruction method which incorporates point spread function (PSF) correction; True-X+TOF = iterative reconstruction method which incorporates point spread function (PSF) correction with incorporated time of flight information

It is important that the images are also examined visually for inconsistencies and artefacts (Figure 6). Visual assessment of reconstructed Jaszczak images at different activity concentrations showed that incorporation of TOF information in reconstruction algorithm substantially improves contrast levels and lowers noise with analytical FBP. FBP showed the lowest levels of contrast and the highest levels of background variability. Iterative reconstruction algorithm (OSEM) and iterative reconstruction algorithm with PSF modelling-True-X produced images with clearly shaped cylinders and spheres with high contrast and low image noise. TOF information had lower impact on improvement of the images reconstructed with iterative reconstruction methods. TOF information showed best results with low activity concentration ratios and less advanced reconstruction methods, where more noise was present.

## Conclusions

The performance characteristics of Siemens Biograph mCT PET/CT scanner were evaluated following the NEMA NU-2-2001 standard, adjusted NEMA NU-2-2001 standard and some additional tests using different methods of topographic reconstruction.. While other studies present either results with NEMA phantoms, or results with in-house-made phantoms, we found it interesting to compare and present both types of the results, which might be applicable in the institutions where NEMA equipment is not available.

All algorithms offered by the Biograph mCT software were included and applied to the wide range of activity concentration ratios. Thus analytical FBP method as traditional reconstruction method was also included into study in order to compare it with modern iterative reconstruction algorithms, which is novelty compared to results performed by other authors.

Our most important interest was in observing the impact of TOF information. On the basis of measurements evaluation we concluded that incorporation of TOF information in the reconstruction algorithm had the greatest impact on background variability reduction, while improvement of spatial resolution is negligible. The comparison of levels of background variability in the vicinity of hot cylinders revealed that they can be higher up to three times compared to background variability in the vicinity of cold inserts for smallest diameters. Lower levels of background variability in the area of spheres could be obtained using separate phantoms for cylinders and spheres. Measurements of relative count error or accuracy of attenuation and scatter corrections showed that TOF correction improved relative count error, especially with higher activity concentration ratios. We observed substantial difference in relative count error for the cases excluding/including the plastic wall. Relative count error measurements should be performed with the same diameter of ROI as the internal diameter of cylinder. When comparing traditional and modern reconstruction algorithms we found out that analytical FBP yields comparable or even better results in some parameter measurements, such as cold contrast and relative count error. Iterative methods show the highest levels of hot contrast, when PSF and TOF correction were applied simultaneously. However, iterative method with PSF modelling produced higher values of relative count error, which can be decreased with implementing TOF corrections. The impact is especially prominent at higher activity concentration ratios. Baseline iterative OSEM (2.8) showed substantially lower levels of background variability than any other reconstruction algorithm, on the other hand, it was inferior in all other parameter measurements.

## Figures and Tables

**TABLE 1.** Measured values of intrinsic spatial resolution in FWHM for various line source positions and reconstruction methods

	**FBP**	**FBP+TOF**	**OSEM**	**OSEM+TOF**	**True-X**	**True-X+TOF**
**1 cm offset (x=0, y=1 cm)**						

**Transverse**	5.2 mm	5.1 mm	4.5 mm	4.5 mm	2.9 mm	2.9 mm
**10 cm offset (x = 10 cm, y = 10 cm)**						

**Transverse radial**	5.9 mm	5.9 mm	4.8 mm	4.8 mm	2.7 mm	2.8 mm
**Transverse tangential**	5.9 mm	5.8 mm	5.3 mm	5.8 mm	3.7 mm	3.9 mm

FBP = filtered back projection; FBP+TOF = filtered back projection with incorporated time of flight information; OSEM = ordered subsets expectation maximization; OSEM+TOF = ordered subsets expectation maximization with incorporated time of flight information; True-X = iterative reconstruction method which incorporates point spread function (PSF) correction; True-X + TOF = iterative reconstruction method which incorporates point spread function (PSF) correction with incorporated time of flight information

**TABLE 2.** Relative count error for various reconstruction methods, performed with regions of interest (ROI) with diameter equal to external diameter of air insert and diameter equal to internal diameter of air insert (in brackets)

	**Ratio 1:2**	**Ratio 1:4**	**Ratio 1:8**

**Reconstruction algorithm**	**ΔC air [%]**		
FBP	11.1 (9.8)	16.2 (14.5)	9.7 (8.4)
FBP+TOF	11.1 (12.1)	12.6 (10.2)	9.8 (8.8)
OSEM	15.0 (15.3)	25.0 (23.6)	21.0 (19.8)
OSEM+TOF	15.3 (15.4)	17.7 (15.3)	14.7 (13.8)
True-X	20.4 (20.5)	25.0 (23.5)	20.3 (18.9)
True-X+TOF	14.1 (14.1)	17.2 (14.7)	14.9 (12.3)
OSEM (2,8)	48.0 (46.9)	45.9 (47.5)	49.1 (47.6)

FBP = filtered back projection; FBP+TOF = filtered back projection with incorporated time of flight information; OSEM = ordered subsets expectation maximization; OSEM+TOF = ordered subsets expectation maximization with incorporated time of flight information; True-X = iterative reconstruction method which incorporates point spread function (PSF) correction; True-X+TOF = iterative reconstruction method which incorporates point spread function (PSF) correction with incorporated time of flight information
